# Supplementary material for: The genome sequence of the hook-banded wasp hoverfly, Chrysotoxum festivum (Linnaeus, 1758)
Source: Wellcome Open Res. 2024 Oct 15;9:448. Originally published 2024 Aug 12. [Version 2] doi: 10.12688/wellcomeopenres.22773.2 (PMC11632216; doi:10.12688/wellcomeopenres.22773.2)
Supplement: Peer review history

# Reviewer report: Reviewer response for version 2

**DOI:** 10.21956/wellcomeopenres.25597.r111054

**Published:** 2024-12-10

**Authors:** İnci Şahin Negiş

**Affiliations:** 1 Selcuk University, Selçuklu-Konya, Turkey

**Genome Sequence Report**

 The presented genome sequence report highlights a highly accurate and reliable genome assembly supported by cutting-edge sequencing technologies and comprehensive analyses. Below are the key findings and implications of the study:

**1. Sequencing Accuracy and Data Quality**

 - Pacific Biosciences single-molecule HiFi long reads achieved a remarkable 99% accuracy, ensuring reliable readings for large and complex genomes. 

 - 800 million reads and 120.81 Gb of sequencing data were generated, providing an exceptionally comprehensive dataset for genome assembly.

 - Hi-C data was utilized to accurately scaffold chromosomes and organize them into a physical map, ensuring the structural correctness of the assembly.

 - With a 139-fold genome coverage, the assembly's precision and reliability were guaranteed.

**2. Genome Assembly Statistics**

 - The estimated Genome Scope heterozygosity percentage(value to be added) complements the observed genome complexity, shedding light on the organism's genetic diversity.

 - A 44% repetitive genome content indicates a significant proportion of transposable elements and other repetitive sequences. This high repeat content suggests the genome's potential role in facilitating rapid adaptation to environmental changes, adding valuable insights into evolutionary biology.

**3. Assembly Quality**

 - A Quality Value (QV) of 61.2 reflects an extremely low error rate, approximately 1 error per 1.3 billion bases, underscoring the assembly's technical excellence.

 - 100% k-mer completeness confirms the assembly's comprehensiveness, ensuring that all sequenced genomic elements are accurately represented.

**4. Biological Completeness**

 - Using the BUSCO v5.4.3 Diptera_odb10 reference dataset (n = 3,285 genes):

   - The assembly successfully contains 96.5% of conserved genes, with 95.5% single-copy genes and 1.0% duplicated genes. While the latter could indicate polyploidy or assembly artifacts, the duplication rate remains acceptably low.

**5. Scientific and Practical Implications**

 - This assembly represents a high-quality resource for studying genome structure, adaptation mechanisms, and evolutionary biology.

 - The comprehensive methodology and robust results significantly enhance the manuscript's scientific value, warranting publication.

 The findings presented in this report demonstrate that this assembly serves as a benchmark-quality genome for *Chrysotoxum festivum* organisms, combining high accuracy, completeness, and biological relevance. The manuscript provides a valuable contribution to the field of genomics and should be published to facilitate further research in this domain.

Are sufficient details of methods and materials provided to allow replication by others?

Yes

Is the rationale for creating the dataset(s) clearly described?

Yes

Are the datasets clearly presented in a useable and accessible format?

Yes

Are the protocols appropriate and is the work technically sound?

Yes

Reviewer Expertise:

molecular entomology, thysanoptera taxonomist

I confirm that I have read this submission and believe that I have an appropriate level of expertise to confirm that it is of an acceptable scientific standard.

# Reviewer report: Reviewer response for version 2

**DOI:** 10.21956/wellcomeopenres.25597.r106927

**Published:** 2024-10-29

**Authors:** Komal Kumar Bollepogu Raja

**Affiliations:** 1 Baylor College of Medicine, Houston, TX, USA

The article by Falk S. and Woodcock K. provides a comprehensive overview of the genome sequencing and assembly of the wasp hoverfly *Chrysotoxum festivum*. The authors present both the biological background of the species and the technical aspects of the genomic work. The methodology is sound and all experimental details are well presented for reproducibility. The assembly metrics are clearly presented, with appropriate benchmarks provided for context. The BUSCO of 96.5% suggests that the genome assembly is high quality. The authors' mention of 524 gaps in the final assembly could benefit from a brief elaboration. Are these gaps expected in this type of genome assembly?

 Overall, the article is well written and the authors present high quality genomic assembly of *Chrysotoxum festivum*. Since not much is known about this hoverfly species, the genome assembly is a great resource for comparative and functional genomic research as well as for ecologists.

Are sufficient details of methods and materials provided to allow replication by others?

Yes

Is the rationale for creating the dataset(s) clearly described?

Yes

Are the datasets clearly presented in a useable and accessible format?

Yes

Are the protocols appropriate and is the work technically sound?

Yes

Reviewer Expertise:

Single cell omics, Genomics, Bioinformatics, Neurobiology

I confirm that I have read this submission and believe that I have an appropriate level of expertise to confirm that it is of an acceptable scientific standard.
